# Development of Antibody Immuno-PET/SPECT Radiopharmaceuticals for Imaging of Oncological Disorders—An Update

**DOI:** 10.3390/cancers12071868

**Published:** 2020-07-11

**Authors:** Jonatan Dewulf, Karuna Adhikari, Christel Vangestel, Tim Van Den Wyngaert, Filipe Elvas

**Affiliations:** 1 Molecular Imaging Center Antwerp, Faculty of Medicine and Health Sciences, University of Antwerp, Universiteitsplein 1, B-2610 Wilrijk, Belgium; jonatan.dewulf@uantwerpen.be (J.D.); christel.vangestel@uantwerpen.be (C.V.); tim.van.den.wyngaert@uza.be (T.V.D.W.); 2 Faculty of Pharmaceutical Biomedical and Veterinary Sciences, Medicinal Chemistry, University of Antwerp, Universiteitsplein 1, B-2610 Wilrijk, Belgium; karuna.adhikari@uantwerpen.be; 3 Department of Nuclear Medicine, Antwerp University Hospital, Wilrijkstraat 10, B-2650 Edegem, Belgium

**Keywords:** positron emission tomography (PET), single-photon emission computed tomography (SPECT), antibody, bioconjugation chemistry, radiopharmaceuticals, pretargeting

## Abstract

Positron emission tomography (PET) and single-photon emission computed tomography (SPECT) are molecular imaging strategies that typically use radioactively labeled ligands to selectively visualize molecular targets. The nanomolar sensitivity of PET and SPECT combined with the high specificity and affinity of monoclonal antibodies have shown great potential in oncology imaging. Over the past decades a wide range of radio-isotopes have been developed into immuno-SPECT/PET imaging agents, made possible by novel conjugation strategies (e.g., site-specific labeling, click chemistry) and optimization and development of novel radiochemistry procedures. In addition, new strategies such as pretargeting and the use of antibody fragments have entered the field of immuno-PET/SPECT expanding the range of imaging applications. Non-invasive imaging techniques revealing tumor antigen biodistribution, expression and heterogeneity have the potential to contribute to disease diagnosis, therapy selection, patient stratification and therapy response prediction achieving personalized treatments for each patient and therefore assisting in clinical decision making.

## 1. Introduction

Continuous progression in the molecular characterization of tumors, biomarker discovery and cancer specific pathways have opened new possibilities for targeted cancer treatment, adding new antibody (Ab)-based treatment options (e.g., trastuzumab, nivolumab,…) to non-specific cytotoxic therapies (e.g., paclitaxel, doxorubicin,…). Ab-based therapeutics in oncology benefit from their high specificity and high affinity for a particular target that is overexpressed on tumor cells, while leaving healthy tissues lacking this target unharmed. Immuno- positron emission tomography (PET)/ single-photon emission computed tomography (SPECT) is defined as the radiolabeling of Abs (Abs) and antibody fragments with positron- (PET) or gamma- (SPECT) emitting radionuclides for imaging. Immuno-PET/SPECT combines the benefits of targeting specificity of monoclonal antibodies (mAbs) with the superior sensitivity of PET and SPECT, which can provide information on whole-body biomarker distribution or tumor target expression in vivo [1]. Functional tumor biomarker changes can be detected earlier with immuno-PET/SPECT, when compared to changes in lesion size as assessed on morphological imaging that typically represent late effects of treatment.

The standard method for biomarker quantification is generally biopsy sampling with downstream immunohistochemistry (IHC) or mRNA validation. This is a suboptimal invasive method compared to immuno-PET/SPECT imaging, since biomarker expression suffers from tumor heterogenicity and subsequent sampling error issues. Additionally, repetitive biopsies and histopathological confirmation are required to monitor treatment response, making clinical use challenging [2]. In contrast, immuno-PET/SPECT can provide a non-invasive, longitudinal and quantitative assessment of tumor target expression and distribution. While liquid biopsies or a rise in serum tumor markers can also identify patients with treatment failure or relapse, subsequent imaging is frequently required to localize the disease and guide subsequent treatment decisions [3]. The current reference standard in routine clinical practice for functional PET/CT tumor imaging is 2-[^18^F]fluoro-2-deoxy-D-glucose ([^18^F]FDG) targeting highly metabolic tissues (tumor, brain, heart,…), but also non-specifically activated inflammatory cells, whereas immuno-PET/SPECT radiotracers show target specific binding. Promising clinical trial data in the setting of HER-2 overexpressing breast cancer support the principle combining baseline target-specific immuno-PET/CT imaging with early assessment of tumor metabolism using [^18^F]FDG-PET/CT to identify the subset of patients who will benefit from the treatment [4].

Ab radiolabeling pioneered with SPECT radio-isotopes (^131^I,^123^I, ^111^In, ^99m^Tc) but interest of the nuclear medicine community shifted along the years towards PET radio-isotopes (^89^Zr, ^64^Cu, ^124^I) since these became more readily available due to optimized nuclear reactions and higher purity. In general, PET scanners produce higher resolution images and have higher sensitivity, resulting in more-accurate image quantification. These benefits are generally offset by higher tracer production costs and higher radiation burden because of higher photon energies of PET radionuclides. In practice, this increased exposure was often balanced since SPECT tracers required higher injected activities due to the lower detector sensitivity [5,6]. However, gradual introduction of novel detector and system designs with higher sensitivity will enable lower administered activities and therefore facilitate immuno-PET/SPECT use in the upcoming years. Another unique characteristic of SPECT radio-isotopes is the ability to perform dual isotope imaging since emitted γ-rays and their accompanying energies are a unique fingerprint of that SPECT radio-isotope. Not only multiple radio-isotope labeled tracers can be imaged using SPECT in this way [7], but they can also be combined with a PET tracer, if combined SPECT/PET hardware is available. Knight et al. took advantage of this property to evaluate the tumor enhanced permeability and retention (EPR) effect using SPECT combined with target imaging using PET in tumor bearing mice [8]. Unfortunately, this technique is currently limited to preclinical research settings.

While several reviews on immuno-PET exist, none provide up-to-date descriptions of immuno-PET and -SPECT imaging with monoclonal Abs in nuclear medicine. After a brief introduction on the monoclonal Ab structure, the different bioconjugation modalities used in immuno-PET/SPECT will be discussed more in depth, ranging from conventional to more recent site-specific strategies. Then an extensive overview of common PET and SPECT radioisotopes used in mAb imaging will be presented, followed by the different radiolabeling strategies, advantages and limitations. Finally, relevant preclinical and clinical studies will be discussed for each radioisotope.

## 2. Antibody Structure

**Figure 1:**
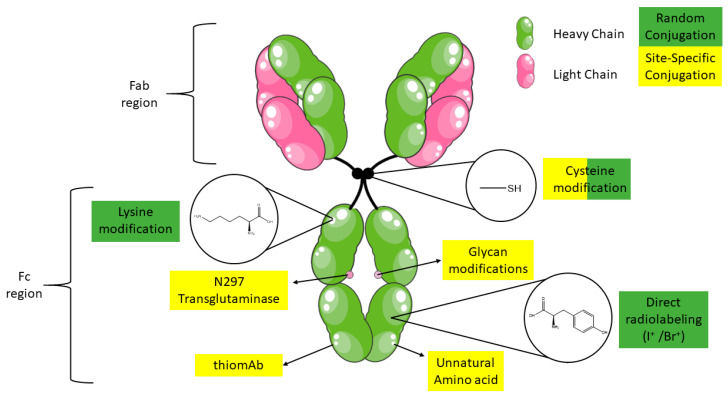
Random and site-specific immuno-PET/SPECT bioconjugation sites of mAb (Figure adapted Servier Creative Commons Attribution https://creativecommons.org/licenses/by/3.0/).

Abs, also known as immunoglobulins, consist of a Y-shaped structure. The bi-fork shaped end comes together in a stalk and both are connected by a flexible hinge region. The fork consists of two distinct functional units: the fragment antigen-binding (Fab) variable region and the stalk consists of the fragment crystallizable (Fc) constant region of the Ab (Figure 1) In human Abs, the Fab region is responsible for antigen recognition, while the Fc region is in charge of the immune response by interaction with effectors cells [9]. There are five classes of immunoglobulins based on the heavy chain constant region: IgA, IgD, IgE, IgG, and IgM. The most abundant Abs in human serum are IgG (80%) and IgM (10%) [10]. Of the five classes, immunoglobulin type 1 Abs (IgGs) are the most frequently used Abs for targeted cancer therapy and cancer immunotherapy. IgGs are about 150 kDa and are composed of two identical polypeptide heavy chains connected with two light chains via disulfide bonds and a conserved glycosylated position at N297 of each heavy chain. IgG are attractive Abs since different anti-tumor mechanisms are available dependent on the chosen immunoglobulin G subclasses (IgG_1_, IgG_2_, IgG_3_ and IgG_4_). For example, IgG_1_ & IgG_3_ allow IgG–Fc-γ-receptors (FcγR) interactions, enabling recognition of these targets by immune effector populations that express FcγR (e.g., NK cells, neutrophils, mononuclear phagocytes and dendritic cells). Cross-linking of FcγRs on these cells promote Ab dependent cellular cytotoxicity (ADCC) and tumor cell destruction [11,12]. On the other hand, IgG_2_ and IgG_4_ are less immunoreactive and are ideal for blocking receptor-ligand/ receptor-receptor interactions. Structural factors are also important in selecting your Ab class e.g., stability of the hinge region (where IgG_1_ = IgG_4_ > IgG_2_ > IgG_3_). The highly unstable hinge region of IgG_3_ is also the reason why this subclass is the only one that has not been explored commercially [13].

In immuno-PET/SPECT binding of the Fc region with Fc receptors for example in the reticuloendothelial system, most notably FcγR can result in off-target sequestration and decrease in quality and diagnostic utility of the images. In a recent immuno-PET study tackling this nonspecific uptake, Ab deglycosylation resulted in higher tumor-to-healthy organ contrast and higher quality PET images [14]. A lower affinity for binding to the FcγR was proposed as mechanism, resulting from the modified 3D configuration of the Fc region [15].

Smaller fragments resulting from biochemical digestion of full-size IgG Abs, such as antigen-binding fragments (Fab, (Fab’)2, Fab’, and scFv), have also been developed for immunoPET/SPECT imaging applications [16]. When compared to full-size Abs all antigen-binding fragments show lower accumulation at the tumor target site, however they benefit from higher tumor permeability and faster clearance from circulation, mainly by the kidneys. Indeed, full-length IgG Abs generally show slow tissue distribution and long circulation time (t_1/2_=1–3 weeks). Slow intracellular catabolism by lysosomal degradation to amino acids after uptake by either pinocytosis, or by a receptor-mediated endocytosis process is the main pathway for IgG elimination. In addition, a salvage pathway mediated by the neonatal Fc receptor (FcRn) exists as a protective mechanism for IgG molecules to maintain their concentrations in the plasma in order to support their physiologic function to provide long-term immunity. The FcRn-mediated recycling releases the IgG molecules back to the vascular space [17]. As a consequence, the elimination half-life for IgG_1_, IgG_2_, and IgG_4_ is ∼18–21 days, which is substantially longer than the half-life of other proteins with similar molecular weight. The long elimination half-life of IgG molecules is a desirable characteristic for therapeutic tumor targeting, since continuous circulation of the Ab will result in maximum exposure and increased accumulation of the Ab at the target site [18].

Recently described radioimmunoconjugates include both full-length IgG mAbs and small Ab fragments. Radiolabeled small fragment derivatives allow obtaining high target to background ratio at earlier time points (1–12 h) following administration, while full-length IgG Abs require significantly greater time periods (1 day–1 week) to enable the Ab to accumulate at the target site and clear from circulation [19]. Ab fragment imaging grants access to the use of shorted lived isotopes (e.g., ^18^F) which generally have more favorable intrinsic properties (higher positron branching ratio (97%), lower average positron energies (250 keV)) and therefore result in better resolution of the PET images. In addition, fragments lack the Fc region, reducing the immunoreactivity and therefore removing activation of effector responses, which is desired in imaging applications. However, conjugation sites on Ab fragments are limited and should be performed carefully to maintain immunoreactivity whereas this risk is minimal in full Abs since more options are available [20]. In addition, labelling intact Abs can still provide useful important longitudinal imaging information, whereas fragments would require repeated injections. Discussing Ab fragments and their applications more into dept lies outside the scope of this paper and therefore the authors recommend some excellent reviews [19,21].

## 3. Radiolabeling and Bioconjugation Strategies of Monoclonal Antibodies

The choice of Ab radiolabeling strategy is influenced by the choice of radioisotope, the nature of the Ab and the availability of the radioisotope. The use of PET and SPECT radiometals, such as ^89^Zr and ^111^In, respectively, requires the use of a bifunctional chelating (BFC) group, while nonmetallic radionuclides, such as ^123^I, can be labeled directly via electrophilic aromatic substitution on histidine or tyrosine amino acid residues on peptides or proteins. In cases where direct labeling conditions are too harsh and thus incompatible with the nature of the Abs, prosthetic groups can be used to circumvent these issues. The conventional prosthetic groups and bifunctional chelators have relied on reactions with natural amino acids for attachment to the Ab structure. These consist typically out of reactions between lysines and N-hydroxysuccinimidyl esters or thiocyanate family and cysteines and maleimides (Michael addition). Since Ab structures consist out of a multitude of cysteines and lysines, heterogeneous mixture of conjugates will be generated which can result in decreased target affinity. To circumvent these issues, site selective reactions were introduced for attaching prosthetic groups. In immuno-PET/SPECT, site-specific conjugation is generally achieved by cysteine engineering, but also new strategies achieving chemoselectivity were developed e.g., click chemistry, enzymatic reactions and biorthogonal transformations (Figure 1).

### 3.1. Conjugation Reactions Using Solvent Accessible Amino Acids in Antibodies

#### 3.1.1. Lysine

A typical IgG1 Ab consists out of 80 lysines from which 30 are highly solvent accessible and therefore easily available for conjugation reactions [22]. A typical conjugation consists of activating the solvent exposed ε-amino groups of the lysine residues on the Ab structure at alkaline pH 9 (more nucleophilic than at neutral pH), by removing the positive charge on these groups. After activation, these lysines can be conjugated using a one-step approach by direct conjugation via an amine reactive group using reactive electrophilic groups such as activated esters, isothiocyanate (SCN), isocyanate, anhydrides, amongst others [23]. Several amine reactive groups have been successfully used in immuno-PET/SPECT and demonstrated adequate stability [24,25]. In mice bearing FaDu human tumors both [^89^Zr]Zr-DFO-p-Phe-SCN-U36 and the [^89^Zr]Zr-DFO-N-SUC-U36 were compared, but no difference in biodistribution/ tumor uptake could be observed [26].

#### 3.1.2. Cysteine

Typically, IgG molecules consist out of 32 cysteine residues forming 4 interchain and 12 intrachain bonds. Only the four interchain disulfide bonds in the Ab hinge region are solvent exposed, and thus accessible for conjugation, creating after their reduction eight reactive thiols [27]. Cysteines in IgG molecules are all in disulfide bonded state, therefore conjugation requires reduction of the disulfide bonds using a reducing agent such as dithiothreitol (DTT) or tris(2-carboxyethyl)phosphine (TCEP). The resulting thiol group of the cysteine residue will typically be reacted with maleimide groups via Michael addition to form thioether bonds. Maleimide conjugated Abs have been widely used and successfully implemented, resulting in adequate tumor visualization in several studies [28,29]. This thioether bond (maleimide reactive group) shows superior in vivo stability over the disulfide bond (pyridyldithiopropionate reactive group) as evaluated in a biodistribution study of [^111^In]In-anti-CEA mAb fragments. The higher renal uptake for S-S conjugated fragments indicates in vivo cleavage of the disulfide bond [30]. However, the thioether bond can undergo a reverse Michael addition reverting back to the free thiol and maleimide starting products, resulting in release of the chelate from the targeting ligand [31]. The free radiolabeled maleimide motif can then undergo in vivo Michael additions with endogenous thiol-containing compounds (e.g., albumin or glutathione) resulting in higher uptake in non-target tissue, reducing uptake in target tissue, increase radiation dose to healthy tissues and reduced imaging contrast [32,33].

Due to suboptimal synthesis and a lack of available alternatives, the maleimide bond remains the standard for cysteine conjugations in immuno-PET/SPECT. Adumeau et al. recognized these in vivo stability concerns, and in an effort to generate a less reactive final bond they developed a simplified synthesis of a new reagent phenyloxadiazolyl methylsulfone (PODS), allowing to move away from the instable maleimide-thioether [28]. Synthesis of the compound was adapted and simplified from Toda et al. [34]. This study compared ^89^Zr-DFO-PODS-huA33 and [^89^Zr]Zr-DFO-mal-huA33 in a PDX model of colorectal cancer and showed similar 50-60% ID/g uptake in the tumors at 48h post injection (pi). However, the stability of [^89^Zr]Zr-DFO-PODS-huA33 was improved when compared to [^89^Zr]Zr-DFO-mal-huA33 [28].

Conventional (non-specific) conjugation can be considered a poor and imprecise manner to conjugate Abs. Three levels of heterogeneity can be observed: number of conjugates attached, regioisomers and batch to batch variability. Undoubtably differences in pharmacokinetic, biological and chemical characteristics are expected between e.g., an Ab conjugate with 1 chelator attached to the Fc region and a construct where 5 chelators are attached to Fab and Fc regions. Sharma et al. evaluated this impact in their search for optimized immuno-PET ^89^Zr radiolabeling [35]. Generally, increasing DFO chelators/Ab resulted in decreased immunoreactive fraction and poor in vivo PET images (increased log D and hepatic uptake, lower radiotracer tumor uptake). All this can act as a barrier to regulatory approval of their clinical application. Comparative studies between random labeling and different site-specific approaches are discussed more in detail below [36].

### 3.2. Site-Specific Cysteine Bioconjugation

In addition to their use in random conjugation, cysteines can also be used for site-specific conjugation. Cysteines are highly interesting compared to other amino acids in proteins, since the nucleophilicity of the deprotonated thiol group exceeds the reactivity of other nucleophilic groups in Abs. Therefore, cysteines have the potential to be engineered in mAbs and selectively conjugated, leaving native residues (disulfide bonds) intact and therefore avoiding potential stability issues (denaturation) observed during disulfide bond reduction [37]. Junutula et al. developed a biochemical assay (phage ELISA for selection of reactive thiols, PHESELECTOR) in which potential sites are identified for mutation to cysteine residues [38].

Tinianow et al. used this assay to develop thiotrastuzumab which contains a cysteine in two regions on the heavy chain of the constant Fab region. In a later stage, these were conjugated via different reactive groups to DFO and radiolabeled with ^89^Zr. Conventional radiolabeling strategies, using random coupling isothiocynate, were compared to these site-specifically conjugated radiotracers in tumor bearing BT474 mice. The number of chelators attached per Ab in the site-specific bioconjugates varied from 1.8–2.0 (showing in some cases incomplete loading of the thiotrastuzumab constructs), compared to 2.4 chelators/Ab when using random conjugation. The affinity to human epidermal growth factor receptor 2 (HER2) was similar in the site-specific modified constructs compared to unbound thiotrastuzumab. Unfortunately, the affinity of the randomly labeled bioconjugates was not assessed. All BT474 tumors could be clearly visualized using radiotracers and showed similar biodistribution and tumor uptake without significant differences between site-specifically and randomly bioconjugated constructs as assessed by ex vivo gamma-counting [29]. Notably, HER2 expressing tumors are known to exhibit irregular growth patterns in mice resulting in heterogeneous target expression tumors and were for this reason abandoned by other groups for proof of concept validation [28].

Using this approach, homogenous well defined bioconjugates could be developed, which provides limited batch to batch variability, which simplifies regulatory approval. However, genetic engineering of the Ab will add additional expenses and complexity to the production process, especially for full mAbs. Maintaining immunoreactivity is another benefit of site-specifically labelling, but in beforementioned study no difference in tumor uptake between randomly and site-specifically labeled bioconjugates could be observed. Full mAbs do contain a large Fc region and no change in immunoreactivity is expected by random conjugation in this region. The engineering and radiolabeling of cysteines in Ab fragments, which generally have fewer bioconjugation site options, resulted in distinct differences in tumor uptake between randomly and site-specifically bioconjugated constructs [39].

### 3.3. Glycans

IgG Abs contain two conserved post-translational modification glycosylation sites that can be chemically modified to enable site-specific attachment of chelators. Glycans are an interesting conjugation target for different reasons: (i) they are located distal to the antigen binding regions, which decreases the potential impact on Ab conjugate immunoreactivity; (ii) their distinct chemical structure grants the potential use of new conjugating methods which is extensively studied and readily available; and (iii) the glycosylation sites (one on each heavy chain) are always located at the Asn297 region, giving access to a natural site-specific moiety. Glycans are however not readily reactive and need to be modified. Three different approaches of glycans modifications have been investigated for immuno-PET/SPECT imaging.

The oldest used method consists of chemical glycan oxidation. The cis-glycol groups of glycans are reacted with periodate (IO_4_^−^) to yield aldehyde groups. These new functional groups are more reactive and can undergo covalent binding with nucleophilic groups (e.g., amines, hydrazide and aminooxy) of chelator containing probes. Formed linkages between nucleophilic groups and aldehydes are not equally stable. Imines (formed by reaction with amines) and hydrazones (formed by reaction with hydrazines) show hydrolytic instability and require an additional reduction step with cyanoborohydride to increase their stability. While hydrazones are more stable then imines, aldehyde oxime ethers (formed by reaction with aminooxy functionalities) show the best stability and do not require an additional reduction step. Using amine bearing reactive groups Rodwell et al. was able to successfully develop [^111^In]In-DTPA-R9.75 and could clearly visualize lymphoma xenografts with an ex vivo tumor uptake of 22% ID/g, 48 h pi [40]. The hydrazine bearing chelator CYT-395 coupled to glycans of a PSMA targeting Ab and radiolabeled with ^99m^Tc was used to visualize prostate cancer in a subset of patients [41]. Another aminoxy nucleophilic group was also successfully used in immuno-SPECT, radiolabeling [^111^In]In-DOTA-trastuzumab site-specifically and targeting HER2 expressing xenografts [42]. In some cases, the harsh oxidizing conditions using IO_4_^−^ can lead to oxidation methionine residues of the Ab, reducing Ab stability. Alternatives to IO_4_^−^ have not yet been explored in the immuno-PET/SPECT setting [43].

To overcome this limitation, the chemo-enzymatic modification of the glycan residues in the Ab was developed. This dual-enzyme strategy consists of removing the terminal galactose residues with β-1,4-galactosidase, followed by insertion of a modified galactose reactive group at this site that will react with a desired chelator using mutant β-1,4-galactosyltransferase (Y289L) [44]. Zeglis et al. was the first to successfully implement this method combined with click chemistry in immuno-PET, using an azide modified galactose. This azide group was then reacted to DFO-modified dibenzocyclooctynes via the strain promoted azide-alkyne cycloaddition (SPAAC) reaction and radiolabeled with ^89^Zr. In PSMA expressing prostate xenograft mice the site-specific-radiolabeled DFO-J591 Ab showed an increased absolute tumor uptake when compared to randomly radiolabeled controls at 96 h pi (68 vs. 58% ID/g) [45].

Using a similar strategy, Kristensen et al. confirmed higher mean tumor uptake values of site-specifically labeled [^89^Zr]Zr-DFO-trastuzumab in nude mice SKOV3 xenografts compared to conventional lysine modifications. Remarkably, the tumor uptake of the glycan modified Ab (24% ID/g) was twice that of the randomly labeled radio-immunoconjugates (11% ID/g) at 120 h pi, while the difference in Kd between both conjugates was not that prominent (1.55 nM vs. 1.45 nM). To investigate whether this rather important difference in tumor uptake was solely due to the difference in immunoreactivity and not because of reduced Fc-FcγR interactions as a result of glycan modifications, a follow-up experiment was conducted. No difference in maximum tumor uptake was found between trastuzumab and deglycosylated trastuzumab when both were randomly radiolabeled, with 11% and 12% ID/g tumor uptake at 120 h pi respectively. This indicates that the high tumor uptake values of the site-specifically modified trastuzumab is related to the antigen binding region [46].

In a recent study, Vivier et al. investigated the influence of site-specifically glycan modified [^89^Zr]Zr-DFO-pertuzumab on Fc-FcγRI binding more in depth, using BT474 xenografts in nude and humanized NOD-SCID mice. In both mouse models site-specifically glycan modified and randomly [^89^Zr]Zr-DFO-pertuzumab labeled conjugates were evaluated, with both showing similar Kd values for HER2. Athymic nude mice carry murine FcγRI and humanized NOD-SCID mice (huNSG) express human FcγRI, showing low and high binding affinity to human Fc part of the Ab pertuzumab, respectively. Ex vivo biodistribution data confirmed similar radiotracer tumor uptake of site-specifically glycan modified and randomly bioconjugated [^89^Zr]Zr-DFO-pertuzumab at 144 h p.i in athymic nude mice. On the other hand, in humanized NOD-SCID animals a significant difference was observed using an identical experimental set-up [47]. This study confirms the importance of site-specific biomodifications in immuno-PET/SPECT and also shows the added benefit of glycan modification in avoiding Fc-FcγR binding, promoting retention in the vascular space and allowing more time for immune interactions.

Another method of site-specific glycan conjugation consists of metabolic cell engineering of the cells that produce Abs. In this approach the cell media is enriched with a specific sugar for insertion into the Ab, relying on the hypothesis that cells will incorporate these into the Ab [48]. Using this method, Rochefort et al. was able to introduce an azide modified mannose onto an anti-CA19-9 Ab, which was reacted with a fluorophore and was able to visualized pancreatic tumors in xenografts [49]. These findings are important for translation to immuno-PET/SPECT, since currently no radiotracers using metabolic glycan engineering are available in clinical routine.

### 3.4. Antibody Engineering

#### 3.4.1. Protein Engineering

Enzymatic modifications resulting in protein engineering of Abs have been around for some years, mostly investigated for fluorescence in vivo applications. However, in immuno-PET/SPECT only a few reports are available that use enzymatic Ab engineering, focusing mainly on sortase A and transglutaminase. The enzymatic bioconjugation via Sortase A cleaves the LPXTG tag in the polypeptide backbone of the protein upon recognition and inserts an oligoglycine tag attached to a probe, which is then radiolabeled in subsequent steps [50]. Successful visualization of HER-2 expressing xenografts was achieved when implementing this approach with Ab fragments [51].

Transglutaminase protein engineering uses transglutaminases to catalyze the isopeptide bond between the acyl functionality of glutamine residues and primary amines. Abs contains many glutamine residues, but a glutamine position (Q295) only becomes accessible for enzymatic modification after deglycosylation (N297 position), due to increased flexibility of the peptide backbone.

Jeger et al. have shown that the site-specific enzymatic modification of mAbs using transglutaminase leads to homogeneous immunoconjugates with defined stoichiometries, which is important for clinical translation. In vivo these immunoconjugates showed higher tumor uptake in a SKOV3ip (derived from ascites cells that developed in a mouse IP cavity injected with SKOV3 cells) human ovarian carcinoma xenograft model, when compared to those prepared using chemical conjugation methods (58.7% ID/g vs. 15% ID/g at 72h pi, respectively) [52]. These findings are remarkable, particular since no difference in immunoreactivity could be observed between chemically (61 ± 20%) and enzymatically (52 ± 2%) modified radioimmunoconjugates. It has to be considered whether the large difference in radiotracer tumor uptake is not solely related to deglycosylation of the Ab, which would result in reduced Fc-FcγR binding (as discussed above). Indeed, when looking at blood % ID/g of the enzymatically and chemically modified Ab (10.19 ± 0.80 vs 2.50 ± 2.23 at 72h pi respectively) such a phenomenon can be hypothesized.

#### 3.4.2. Unnatural Amino Acids (uAA)

**Figure 2:**
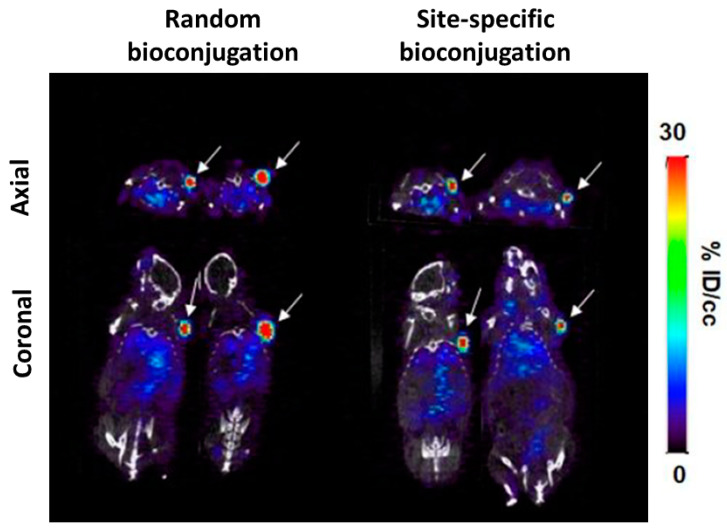
Single slice PET/CT images of random (left) and site-specific unnatural amino acid pAMF-DBCO SPAAC (right) conjugated [^89^Zr]Zr-DFO-Trastuzumab 96h post injection. Adapted with permission from Ahn et al. [53]. Copyright 2020, American Chemical Society.

The incorporation of unnatural amino acids in Abs is challenging and not yet fully explored in immuno-PET/SPECT applications. This approach consists out of expanding the genetic code of the cells, during the translation phase of the recombinant Ab, to interpret nonsense codons as uAA, leading to incorporation of the UAA in the Ab. Using a cell-free method, Boros and coworkers were able to insert four para-azidomethyl-L-phenylalanine (pAMF) residues on the heavy chain of the Fc region of trastuzumab, which was at a later stage conjugated with DFO-PEG_4_-DBCO via SPAAC for [^89^Zr]Zr^4+^ radiolabeling. In SKOV3 tumor bearing mice site-specifically labeled [^89^Zr]Zr-DFO-trastuzumab showed slightly higher radiotracer tumor uptake 33.50 ± 8.48% ID/g when compared with conventional random (p-SCN-DFO) lysine conjugated radiolabeling 31.86 ± 7.97% ID/g at 96 h pi [53] (Figure 2).

Undoubtedly the authors were able to create homogenous site specifically labeled radioimmunoconjugates using unnatural amino acids, facilitating possible future regulatory approval of the conjugates. In the site-specific conjugated constructs 4 chelators/Ab could be observed, whereas the randomly labeled approach showed binding of 0–5 chelators/Ab. However, in their concluding experiments only a trend towards significance over the randomly labeled approach could be established. In addition, the cell binding assay showed no difference in immunoreactivity between radioimmunoconjugates. Stability between constructs was similar but notably in this study ex vivo bone uptake of all conjugates was negligible with < 3% ID/g at 96 h pi, whereas DFO is known to show instability in vivo in mice. These findings are probably related with premature [^89^Zr]Zr-DFO cleavage of the Ab resulting in fast < 24 h [^89^Zr]Zr-DFO clearance via the kidneys, a possible confounder to their results [54]. Certainly, this site- specific approach is the most challenging and implementation will require considerable resources.

### 3.5. Pretargeting Approach

Pretargeting is a commonly used approach to circumvent the limitations of directly radiolabeled Abs. These normally show long circulation times and can therefore increase the radiation dose to healthy tissues during immuno-PET/SPECT imaging.

**Figure 3:**
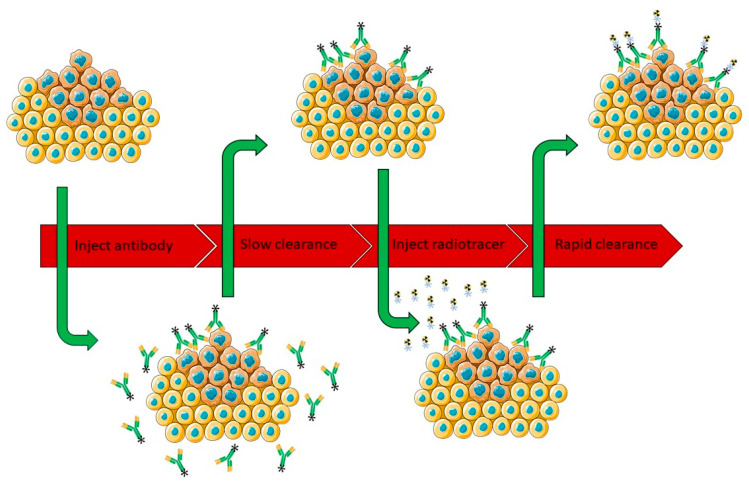
Pretargeting approach: In a first step an Ab with a targeting moiety is injected. Secondly the Ab is allowed to accumulate at tumor site over time. Thirdly a complementary radiolabeled probe is injected and lastly in vivo ligation of the Ab and the radioligand followed by rapid clearance of any excess of the radioligand (figure adapted under a Servier Creative Commons Attribution https://creativecommons.org/licenses/by/3.0/).

In a pretargeting approach the Ab is initially conjugated to a targeting moiety and allowed to accumulate in the Ab target before a complementary, fast clearing small radiolabeled probe is injected. This probe will find and react specifically to the targeting moiety present in the Ab bound to the target, while the unreacted probe is cleared fast, enabling high target-to-background ratio (Figure 3) [55,56]. Separate injections of radionuclide and Ab have several benefits. On the one hand it decreases circulation time of radioactivity, reduces the uptake of the radioisotope in healthy tissue and therefore lowers the radiation dose for the patient. On the other hand, it facilitates the use of short-lived radionuclides, opening up new perspectives.

The nature of the different pretargeting approaches is similar and several mechanisms for pretargeting are available: the use of bi-specific Abs binding a tumor specific antigen and radiolabeled haptens, avidin-biotin systems, complementary oligonucleotides and biorthogonal reactions [57,58,59,60,61,62]. An excellent review describing these different mechanisms has already been published by Altai et al. [63]. Although all of these strategies have proven to be effective, biorthogonal click reactions have recently emerged as a promising tool for in vivo pretargeting due to their favorable properties such as biorthogonality, fast reaction rates and selectivity. First generation bioorthogonal reactions, such as the Staudinger ligation and the SPAAC, suffered from sub-optimal reaction kinetics which made them unsuitable for in vivo pretargeting applications [64,65].

The revisiting of the inverse electron demand Diels-Alder (IEDDA) biorthogonal reaction, achieved between a 1,2,4,5-tetrazine (Tz) and *trans*-cyclooctene (TCO), by several research groups however boosted the pretargeting immuno-PET/SPECT approach. Apart from its selectivity and biorthogonality, the speed plays a pivotal role in the feasibility of in vivo pretargeting, with the first order rate constant between a TCO and tetrazine being of 10^5^ M^−1^s^−1^, which is orders of magnitude faster than either the Staudinger or SPAAC ligations [66]. The vast majority of IEDDA-based pretargeting uses a TCO-labeled Ab and a tetrazine based radioligand [66]. Robillard et al. are the pioneers in the use of IEDDA in pretargeting approach. In their initial work, a TCO-Ab conjugate (CC49-TCO) and an ^111^In labeled DOTA-tetrazine radioligand was employed for the SPECT imaging of TAG-72 expressing colorectal cancer xenografts with excellent image contrast [67]. Additionally, pretargeting studies of TCO-5B1-Ab targeting carbohydrate antigen 19.9 on pancreatic cancer xenografts with ^64^Cu-labeled tetrazine showed that this approach is viable even in the difficult circumstances presented by a circulating antigen and internalized targeting vector [68]. The pretargeting approach further expanded the immuno-PET scope with even shorter-lived isotopes such as ^68^Ga and ^18^F. Using TCO-cetuximab and ^68^Ga-labeled tetrazine epidermal growth factor receptor (EGFR) expressing xenografts could be clearly visualized [69]. Similarly, using TCO-5B1 and ^18^F-tetrazine pancreatic cancer xenografts could be imaged [70]. Pretargeting studies often involve radiolabeled tetrazine compared to its counterpart TCO for which the radiolabeling is less explored, due to the in vivo instability and possible non-specific uptake related to high lipophilicity of TCOs. Fox and co-workers were the first to describe a ^18^F-labeled TCO probe based on classic trans-cyclooctenol [71]. However, ^18^F-TCO was shown to degrade rapidly in vivo giving rise to radiometabolites [72]. Recently, more reactive ^18^F-labeled conformationally strained TCOs were described with better stability profiles [73,74]. Of these, only cis-dioxolane fused transcyclooctene (dTCO) has been successfully evaluated in SKOV3 xenografts, with the ability to clearly visualize the tumor [75].

Zeglis et al. initiated a study where the promising IEDDA pretargeting approach and directly radiolabeled Ab were compared. Using SW1222 xenografts injected with direct labeled [^64^Cu]Cu-NOTA-A33 yielded 33% ID/g absolute tumor uptake. In the pretargeting approach SW1222 xenografts bearing mice were firstly injected with TCO modified Ab and after 24 h with their radioactive counterpart 64Cu-NOTA-Tz and showed 4.0% ID/g tumour uptake at 24 h post injection. However, the tumor to blood ratio of these constructs was higher in the pretargeting approach (2.9 vs. 1.9 at 24 h pi). However, at 48h pi the tumor to blood ratio for [^64^Cu]Cu-NOTA-A33 was increased to 24.5 surpassing the maximum ratio for the pretargeting approach. An additional study with [^89^Zr]Zr-DFO-A33 showed similar results as ^64^Cu labeled mAb in comparison with the pretargeting approach. Final dosimetry calculations showed superiority of the pretargeting approach with an effective dose 0.0124 mSv/MBq compared to [^89^Zr]Zr-DFO-A33, 0.416 mSv/MBq and [^64^Cu]Cu-NOTA-A33, 0.0359 mSv/MBq [76].

A challenge in the pretargeting approach is slow clearance of the modified Ab. Ideally injection of the complementary counterpart is done when Ab accumulation at the tumor site has reached a maximum. However, additional waiting time is necessary to allow the Ab to clear from circulation. Otherwise high background, due to binding of the radiolabeled probe to the Ab in circulation, would result in suboptimal images. Increasing the waiting time before injecting the radiolabeled probe could result in internalization of the Ab, resulting in decreased available secondary moiety. The use of a three step pretargeting approach, where a clearing agent is administered before injection of the complementary counterpart showed improvement in aforementioned problem. However, when administering a clearing agent some considerations have to be made regarding the reduced amount of Ab in the target, immunoreactivity and toxicity. Translation to clinic using this approach is not straightforward either with three different injections and two return visits [77].

Internalization of the Ab resulting in decreased available secondary moiety is another concern of the IEDDA pretargeting approach, specifically since almost all Abs internalize, though at different rates. The opposite is true for direct labeled Abs, where internalization is an advantage resulting in accumulation of radioactivity in the tumor. A rule of thumb suggests that vectors for pretargeting should not be internalized upon binding their target, therefore researchers are limited to certain targets (tumor-associated glycoprotein 72 – A33) to test their pretargeting proof of concept [78,79]. However recent reports indicate that pretargeting is possible with internalizing systems. Keinänen et al. were able to successfully visualize A431 and BT-474 xenografts using internalizing TCO-trastuzumab and TCO-cetuximab, respectively. In this approach TCO-trastuzumab and TCO-cetuximab were coupled with an average of five and six TCO moieties respectively and injected 72 h post radioactive [^18^F]TAF injection. PET imaging was obtained 4 h post radioactive tetrazine injection. Ex vivo biodistribution of pretargeted TCO-trastuzumab showed 1.38 ± 0.20% ID/g and TCO-cetuximab showed 3.70 ± 0.13 tumour uptake [80].

## 4. Radionuclides for Antibody Imaging

Table 1 is an overview for all the radionuclides discussed below.

### 4.1. Common Radionuclides

#### 4.1.1. Iodine

Radioiodination was the first immuno-PET/SPECT (^124^I/^123^I) imaging and theranostic (^131^I) application. The process usually involves the action of a strong oxidizing agent to transform iodide ions into a highly reactive electrophilic iodine which then reacts via electrophilic substitution on the phenolic group of the Ab tyrosine. There are three main iodination protocols: (i) Bolton-Hunter; (ii) based on chemical oxidation of iodide with chloramine-T; and (iii) based on enzymatic oxidation of iodide with lactoperoxidase. All these methods can yield radiolabeled Abs with high specific activities.

Although full-length Abs have been successfully radio-iodinated using these methods, in some cases, the use of strong oxidizing agents, such as chloramine-T, can lead to unwanted oxidizing reactions of the Ab (SH group), reducing the Ab stability [83]. Milder reaction conditions using the lactoperoxidase method have been introduced, in which the enzyme catalyzes the oxidation of iodide by using hydrogen peroxide [84]. In addition, newer techniques aim to limit the access of the oxidizing agent to Ab reactive functionalities by using solid phase oxidation (Iodogen) or a two-step approach (Bolton-Hunter) [85,86]. The formation of a radioiodinated Ab structure containing a phenolic group and iodine atom in the ortho position, using the abovementioned methods, may increase susceptibility for deiodination of the Ab due to deiodinase enzymes involved in thyroid hormone metabolism.

The *N*-succinimidyl 4-iodobenzoate (SIB) based method (derived from Bolton-Hunter), addresses this problem. The method consists of labeling the *N*-succinimidyl (tri-*n*-butylstannyl) benzoate precursor with radioactive iodine via iododestannylation and further on attaching the complex randomly to lysine residues of the Ab. Differences between both labeling strategies are the absence of a hydroxyl group and the location of iodine in the para position. Indeed, in a biodistribution study comparing both chloramine-T and SIB radiolabeling methods in human melanoma xenografts in nude mice has shown decreased thyroid uptake for an Ab radiolabeled using the SIB method, indicating improved in vivo stability [87].

Once the Ab-antigen complex is internalized, it is generally rapidly taken up within lysosomes. Ab catabolism in these lysosomes increases the clearance of iodotyrosine, uptake in the thyroid and therefore resulting in low tumor to background ratios [88]. This can be overcome by using a non-hydrolysable dilactitol-tyramine (DLT) linker coupled with radioactive iodine, which keeps it inside cells once the Ab-antigen complex is internalized resulting in increased tumor/blood and tumor/organ ratios when compared to chloramine-T [89].

In a more recent study radio-iodinated BODIPY was investigated as a prosthetic group for nuclear and optical dual functional labelling agent of trastuzumab. [^123^I]I-BODIPY-trastuzumab was evaluated in HER2-positive N87 tumor bearing mice. SPECT/CT images revealed clear visualization of the tumor and importantly showed no thyroid radioactivity accumulation, indicating adequate stability of the radiotracer [90].

Ab radiolabeling was pioneered with immuno-PET (^124^I) and -SPECT(^123^I) tracers, with more than 60 years of research, making translation in humans possible. In a subset of patients with metastatic colorectal cancer 83% of the primary lesions were detected by [^124^I]I-huA33 Ab immuno-PET. All liver and 57% nodal metastasis could be detected by [^124^I]I-huA33 scans [91]. Delaloye et al. was able to successfully visualize 86% of the known tumor sites with [^123^I]I-anti-CEA immuno-SPECT [92]. A theranostic approach with ^131^I has been evaluated in patients as well with [^131^I]-Tositumomab, an anti-CD20 mAb targeting lymphoid malignancies which received FDA approval in 2003 [93].

#### 4.1.2. ^76^Bromine

The high positron branching ratio β^+^ (55%) is the main advantage of ^76^Bromine (t_1/2_ = 16.2 h) resulting in increased image sensitivity whereas in other radionuclides this is remarkably lower (^124^I; 23%, ^89^Zr; 23%). Unfortunately, the emitted positrons are highly energetic which generates decreased intrinsic spatial resolution and can raise radiation dose concerns. In addition, the half-life of ^76^Bromine is suboptimal for Ab imaging, with other better alternatives available (^124^I; 4.2 days and ^89^Zr; 3.3 days). Besides the aforementioned challenges the major problem of ^76^Bromine is the availability, large scale production and expensive enriched ^76^Se targets resulting in lack of extensive and additional research.

Direct radiobromination of the Ab can be performed in similar manner as radioiodination [94]. However, due to the lower oxidation potential of ^76^Br suboptimal yields are achieved, which implies the use of more powerful oxidizing agents, at the risk of further impacting Ab stability and immunoreactivity. Therefore, an indirect radiolabeling approach was developed, where the initial precursor molecule was radiolabeled with ^76^Br and subsequently coupled to the Ab via amine groups on lysine residues. Several precursor molecules have been investigated: succin-imidyl-3(4-hydroxypentyl) propionate (SHPP), N-succinimidyl bromobenzoate and 7-(*p*-isothiocyanato-phenyl)dodecahydro-7,8-dicarba-nido-undecaborate(1-) ion (NBI) [95,96]. The highest yield (56%) was obtained using NBI as precursor [97]. While all prosthetic groups described above resulted in increased immunoreactivity of the Ab compared to direct radiobromination only NBI radiobrominated Abs showed cellular retention. Reduced enzymatic debromination and radioactive exocytosis of this prosthetic group is related to the molecular structure which is completely foreign to the body. Since iodine and bromine have similar chemical properties it is hypothesized that the similar tracer metabolization and excretion pathways take place [98].

In vivo studies of ^76^Brominated Abs have been successfully performed. A comparative PET imaging study comparing ^76^Br-labeled anti-carcinoembryonic antigen mAb [^76^Br]Br-38S1, [^18^F]FDG and L-[methyl-^11^C]methionine ([^11^C]Met) in human colon carcinoma bearing rats showed that [^76^Br]Br-38S1 was the superior imaging agent, with higher tumor/tissue ratios [99,100]. However, translation to clinical trials with brominated Abs are limited or even non-existent, possibly the result of the combined challenges discussed in the first paragraph when using ^76^Bromine.

#### 4.1.3. *^89^*Zirconium

^89^Zirconium, with a half-life of 3.3 days, is a suitable radioisotope for Ab imaging, since the half-life matches the clearance of the biological vector. It decays to ^89m^Y via β^+^ (23%) and electron capture (EC) with additional decay to ground state via γ rays (100%, 909 keV) [101]. ^89^Zr offers higher quality PET images because of its more favorable β^+^ energies when compared to ^124^I. ^89^Zr can be produced easily and is available worldwide with high purity >99.9%.

**Figure 4:**
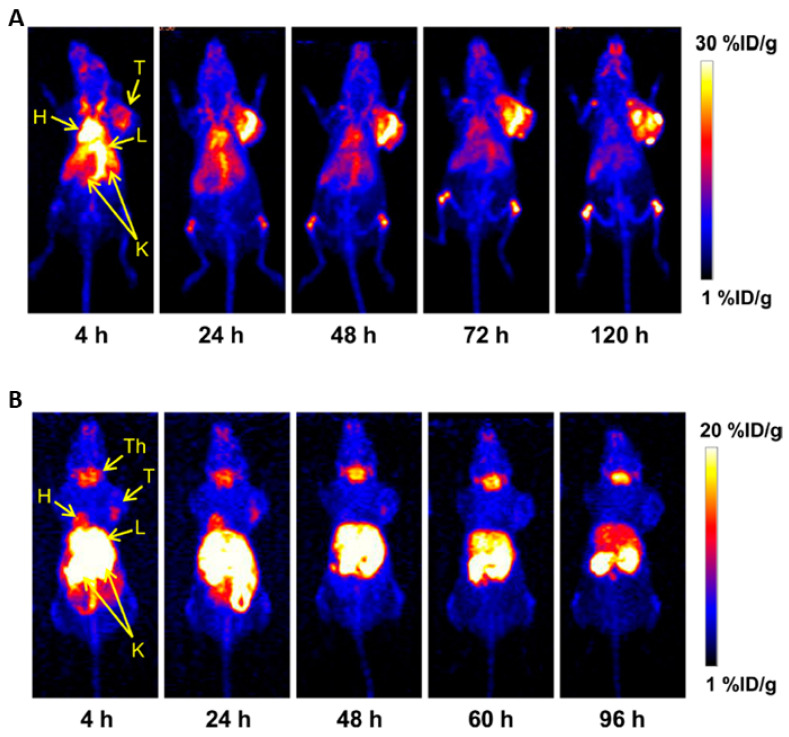
Serial maximum intensity projection (MIP) PET images of mice injected with (**A**) [^52^Mn]Mn-DOTA-TRC105 and (**B**) ^52^MnCl_2_. Note: H, Heart; L, Liver; K, Kidneys; T, Tumor; Th, Thyroid. Adapted with permission from Graves et al. Copyright 2015 American Chemical Society [149].

The major difference and benefit of ^89^Zr over the beforementioned radionuclides is the residualizing characteristic, meaning that it becomes trapped in the target cells after Ab internalization, whereas non-residualizing ^124^I (or ^76^Br) is excreted as iodotyrosine from the tumor cells when directly labeled to the Ab (Figure 4). In a comparison study between ^89^Zr and ^124^I radiolabeled chimeric mAb U36, ^124^I-U36 (16% ID/g) was outperformed by [^89^Zr]Zr-DFO-U36 (23% ID/g) with respect to tumor uptake at 72 h pi This residualizing characteristic increased the overall tumor/ background ratio and thus image quality. However, depending on the biodistribution of the antibody, residualizing of ^89^Zr may occur throughout the body and specifically in clearance organs (liver) resulting in an overall higher background activity [102].

Up to now, the most commonly used chelator for ^89^Zr is desferrioxamine (DFO) and is still heavily used due to lack of other affordable and commercially available alternatives in the past. In general, the BFC is attached at random to the Ab, since this method is the easiest achieved and can be radiolabeled in neutral pH at acceptable temperature (37 °C) with high yield (>98%), excellent purity (>99%) and good specific activity (5 mCi/mg) [103]. However, in vivo implementation of the [^89^Zr]Zr-DFO-Ab tracers showed instability of the DFO complex. Non-chelated ^89^Zr accumulates in bone, with values that reach >10% ID/g, 5 days pi. The effect is more pronounced in internalizing Abs, which suggests a role for catabolism of the [^89^Zr]Zr-DFO-Ab in the cell and removal of ^89^Zr, transchelated to proteins, out of the cell. Transchelation of ^89^Zr to plasma proteins is mentioned as well as possible cause of high bone uptake [2]. Remarkably, the unwanted bone uptake seems to be prominent in rodents as it is hardly an issue in patients [104].

The development of a new chelators DFO* and cycloDFO*, with four hydroxamate groups, showed reduced femur uptake in a comparison study in SKOV3 tumor bearing mice to DFO with 1.5% ID/g, 2.0% ID/g and 4.5% ID/g for [^89^Zr]Zr-cycloDFO*-trastuzumab, [^89^Zr]Zr-DFO*-trastuzumab and [^89^Zr]Zr-DFO-trastuzumab 168h pi respectively [105]. Using another linker, desferrioxamine squaramide (DFOsq) showed better results compared to NCS-DFO, reducing bone uptake but not as pronounced as using chelator DFO* [106]. 3,4,3-(LI-1,2-hydroxypyridinone) (HOPO), which contains hydroxypyridinone groups instead of hydroxamate groups showed in vitro less favorable complex stability then DFO but outperformed DFO in vivo with 2.4% ID/g bone uptake compared to DFO, 17.0% ID/g at 14 days pi [107]. In a recent study using a mini-explorer total body PET system researchers were able to scan 30 days post injection in rhesus monkeys with ^89^Zr radiolabeled Abs, and therefore opening new possibilities of disease monitoring [108].

^89^Zr-immuno-PET tracers have been extensively translated into the clinic, with numerous examples available. The most well-known examples are [^89^Zr]Zr-DFO-trastuzumab and [^89^Zr]Zr-DFO-bevacizumab. Trastuzumab binds to HER2 receptors, which are expressed in breast cancer. Of breast cancer patients only 20–30% are HER2 positive and only these will benefit from Herceptin^®^(trastuzumab) treatment. [^89^Zr]Zr-DFO-trastuzumab has proven to be a reliable biomarker in oncology. In a subset of HER2 negative primary breast cancer patients [^89^Zr]Zr-DFO-trastuzumab was able to detect unsuspected HER2 positive metastases in 15% of the patients [109]. Also, in breast cancer patients with unclear HER2 (positive/negative) status after normal evaluation with [^18^F]-FDG PET-scan, [^89^Zr]Zr-DFO-trastuzumab immuno-PET could assist physicians in treatment planning [110]. Additionally, [^89^Zr]Zr-DFO-trastuzumab was shown to be useful in response prediction of patients treated with TDM-1 (trastuzumab-emtansine antibody drug conjugate) [4].

The target of bevacizumab is VEGF-A, which is overexpressed in different tumor types and is involved in the development and maintenance of tumor angiogenesis. In primary breast cancer [^89^Zr]Zr-DFO-bevacizumab was able to image 25 of 26 breast tumors [111]. In patients with neuroendocrine tumors [^89^Zr]Zr-DFO-bevacizumab was able to detect a treatment effect of everolimus, with a 35% tumor SUV_max_ decrease [112]. An additional pilot study in 7 NSCLC patients showed a SUV_peak_ that was 4 times higher than in normal tissues [113].

The PD-1/PD-L1 axis has been shown to induce anti-cancer immunity when blocked by Abs. Clinical trials with anti-PD-1 and anti-PD-L1 have shown impressive results in heavily treated cancer patients, rekindling the interest of immunotherapy in oncology [114]. However, not all patients benefit from these treatments which results in sometimes rather low response rates, combined with high treatment costs. ^89^Zr-immuno-PET scans for baseline and response assessment may be beneficial in patient selection and predicting treatment success. Clinical trials evaluating [^89^Zr]Zr-DFO-nivolumab (targeting PD-1) are currently ongoing but in an initial study of [^89^Zr]Zr-DFO-nivolumab and [^18^F]F-anti-PD-L1 adnectin whole body PET/ CT scans correlated with tumor PD-1 and PD-L1 expression as assessed by IHC [115]. In addition Bensch et al. reported in an [^89^Zr]Zr-DFO-atezolizumab (anti-PD-L1) study in patients treated with atezolizumab a better predictive value in tracer uptake then IHC as treatment response [116].

Long circulation times of ^89^Zr-mAbs accompanied with high energy γ-rays require careful assessment to limit radiation related toxicity. For example, an early phase [^89^Zr]Zr-trastuzumab study reported a whole body effective dose of 0.47 mSv/MBq whereas [^18^F]F-FDG PET reported a mean effective dose of only 0.0199 mSv/MBq [6,117]. More interestingly, using [^68^Ga]Ga-NOTA-HER2-nanobody resulted in an effective dose of only 0.043 mSv/MBq [16].

#### 4.1.4. *^64^*Copper

^64^Copper a with half-life of 12.7 h, decays to ^64^Zn via β^−^ (38%) and to ^64^Ni via β^+^ (18%) and EC (44%). While its half-life is too short for Ab imaging and more suited for Ab fragment imaging, it has been actively investigated, as its dual β emission makes ^64^Cu a good candidate for both PET imaging and therapy [118,119]. In addition, since the ^64^Cu emits lower positron-energy than other radionuclides discussed here, e.g., ^66^Ga and ^89^Zr, ^64^Cu-immunoPET images exhibit better resolution with high quality [120].

Numerous chelators for [^64^Cu]Cu^2+^ complexation are available, as reviewed by Price et al. who concluded that 1,4,7-triazacyclononane-1,4,7-triacetic acid (NOTA) is the current reference standard and suitable for mAb imaging. New chelators are still being developed but most conventional chelators have acceptable in vivo stability within the 48 h pi timeframe desired for ^64^Cu imaging [121].

Even though the half-life of ^64^Cu (12.7 h) is not ideal for antibody radiolabeling it has been investigated in patients, mostly using [^64^Cu]Cu-DOTA-trastuzumab. Mortimer et al. was able to show strong correlation between [^64^Cu]Cu-DOTA-trastuzumab uptake and HER2 status [122].

#### 4.1.5. *^86^*Yttrium

^86^Yttrium (t_1/2_ = 14.7 h) has high energy β^+^ (32%) decay, accompanied by high energy γ-rays (139–4900 keV) which shows less favorable intrinsic characteristics than those of ^89^Zr for immuno-PET imaging. Similar to ^64^Cu, the half-life seems short for imaging of large mAbs, and thus more suitable for imaging mAb fragments. Despite the available diagnostic/therapeutic ^86^Y/^90^Y matched-pair (which allows the use of same chelator) for radio-treatment and PET follow up interest in clinical practice is very limited, mostly due to the unfavorable half-life and high energy gamma-rays [123].

Diethylenetriaminepentaacetic acid (DTPA) is one of the first generation chelators and has been successfully used to complex yttrium to mAbs [124]. Unfortunately, suboptimal in vivo stability of these radiolabeled Abs was observed [125]. This has encouraged the research community to develop new chelators for this radiometal, such as cyclohexyl-diethylenetriaminepenta acetic acid (CHX-A″-DTPA) that has been used in multiple ^86^Y immuno-PET studies with good stability [126]. However, the modified chelator is still considered less stable in vivo compared to DOTA [125]. While CHX-A″-DTPA and DOTA show good stability, labeling generally requires mild heating (40–60 °C) and long reaction times (30–60 min). Other new chelators, 5p-C-NETA and H_4_octapa have also been developed with promising stabilities for yttrium radiolabeling of Abs [127,128].

Currently no in human studies with ^86^Y-mAb are available. However, the radiometal is heavily studied in preclinical work. Nayak et al. was able to visualize EGFR expressing xenografts of colorectal, prostate, ovarian and pancreatic tumors using [^86^Y]Y-CHX-A’’-DTPA-cetuximab [129]. Generally, ^90^Y-immunotherapy dose limiting studies are calculated based on ^111^In-mAb biodistribution in clinical setting. However, a preclinical comparison study between [^86^Y]Y-CHX-A’’-DTPA-hu3S193 and [^111^In]In-CHX-A’’-DTPA-hu3S193 Abs in HCT-15 xenograft mice did show differences in distribution, related to the different radiometal attached. Therefore, calculating absorbed dose estimates and exposure based on ^111^In-mAb would result in inaccurate values [130]. In addition, the PET diagnostic/therapeutic ^89^Zr/^90^Y pair was evaluated in a clinical setting and ^89^Zr-mAb was able to predict biodistribution and dose limiting organ toxicity of the ^90^Y-mAb [131]. However, ^86^Y as a surrogate for ^90^Y seems to be more suitable than ^89^Zr since the uptake of ^89^Zr-cetuximab in the bone was higher than that of ^86^Y-cetuximab [132].

#### 4.1.6. *^111^*Indium

Over the past decades Indium (^111^In) has been the reference standard in immuno-SPECT imaging of Abs. ^111^In (t_1/2_ = 2.8 days) which decays by EC (100%) and low energy γ-rays emission (171 keV, 245 keV), has shown more favorable characteristics for in vivo Ab imaging compared to frequently used ^123^I in half life and to ^131^I in imaging quality [82]. In a comparison study 5 patients received both [^131^I]I-anti-CEA and [^111^In]In-anti-CEA in which [^111^In]In-anti-CEA could confirm 87% of the tumor lesions and [^131^I]I-anti-CEA only 53%. In addition tumor/background ratio was considerably higher for [^111^In]In-anti-CEA SPECT scans [133]. In addition, when comparing [^111^In]In-DTPA-trastuzumab and [^89^Zr]Zr-DFO-trastuzumab in SKOV3 xenograft similar tumor uptake (39.3% ID/g vs 33.4% ID/g) 6 days pi and similar biodistribution could be observed except for the bone. However, the spatial resolution provided by [^89^Zr]Zr-DFO-trastuzumab was unattainable with [^111^In]In-DTPA-trastuzumab [2].

The chelators DOTA, DTPA and the derivative CHX-A’’-DTPA can be complexed with [^111^In]In^3+^ and all bioconjugates show good in vivo stability. DTPA is generally considered as reference standard for ^111^In immuno-SPECT radiolabeling, while the use of CHX-A’’-DTPA in the preclinical setting is limited. The optimal chelator to use with ^111^In remains to be identified [134].

The clinical translation of ^111^In-labeled Abs has been successful in several types of human cancer. [^111^In]-trastuzumab was evaluated in HER2 positive breast cancer patients where it showed a detection rate of 45% [135]. [^111^In]In-J591, a mAb targeting PSMA in the neo-vasculature of non-prostate solid tumor, was able to detect 74% skeletal, 53% nodes and 64% soft tissue/organ lesions [136].

#### 4.1.7. *^99m^*Technetium

The development of Abs labeled with technetium has been facilitated by the easy availability of this radionuclide from commercial ^99^Mo/^99m^Tc generators. The intrinsic characteristics of ^99m^Tc except for the half-life (t_1/2_ = 6 h) are favorable for immuno-SPECT with 99%, 140 keV γ emission. Owing to its stable and readily accessible oxidation states (between −1 and +7), ^99m^Tc has a versatile chemistry, making it possible to produce a variety of complexes with specific desired characteristics, which is a major advantage for Ab conjugate development. The multiple oxidation states are characterized by chemically robust core structures that can be exploited as platforms for the labeling of Abs with technetium [137].

Both indirect and direct radiolabeling strategies have been investigated with ^99m^Tc. Direct radiolabeling is done by first reducing the disulfide bonds of the Ab. These free thiols are then radiolabeled by transchelation of ^99m^Tc from ^99m^Tc-glucoheptonate or ^99m^Tc-methylene diphosphonate [138]. However, reducing these bonds may lead to Ab aggregation and degradation. Additionally, the reducing agent SnO_2_, required for reducing ^99m^Tc, can reduce functional groups on the Ab, which results in even more stability issues or reduced immunoreactivity [139].

An indirect way of radiolabeling ^99m^Tc is by using bifunctional chelators. DTPA and hydrazine nicotinamide (HYNIC) have been conjugated and radiolabeled successfully to Abs [140,141]. A comparison study between direct and indirect (e.g., using [^99m^Tc]Tc-DTPA/ [^99m^Tc]Tc-HYNIC labeled Abs) radiolabeling methods showed better Ab stability when using indirect methods [142,143]. However, comparing both methods in vivo with immuno-SPECT reference standard ^111^In-labeled Abs only resulted in similar observations with the [^99m^Tc]Tc-HYNIC labeled Abs, indicating the superiority of the [^99m^Tc]Tc-HYNIC over the DTPA labeling method [143].

Indirect radiolabeling of Abs with ^99m^Tc is an improvement over direct labeling but the method still suffers from random binding of ^99m^Tc (without chelator), formation/binding of colloids to the Ab and difficulties in controlling the oxidation state of ^99m^Tc. Complexation of ^99m^Tc to a (diamide dithiolate) N_2_S_2_ ligand (e.g., 4,5-bis(thioacetamide)pentanoate) prior to conjugation to the Ab can remove these issues. This ligand is functionalized with a COO^−^ group, which can be activated by an ester group and attached to the lysines of the Ab in similar manner as described before [144].

^99m^Tc-radiolabeled Abs have been successfully translated to patients. In a subset of 10 patients with non-Hodgkin’s lymphoma (NHL) a moderately increase in [^99m^Tc]Tc-rituximab (anti-CD20) activity could be observed in all but one CT-scan confirmed NHL [145]. In another clinical trial using anti-EGF-r radiolabeled Ab [^99m^Tc]Tc-h-R3 could a specificity of 100% and a sensitivity of 76.5% be observed [146].

### 4.2. Emerging Radionuclides

In this part of the review paper some emerging radionuclides are highlighted. The studies mentioned here represent for some of these the first data on radiolabeling, chelator, stability and immuno-PET/SPECT imaging. Some of them show interesting characteristics as discussed below but additional research and comparison studies will be required to conclude if these emerging radionuclides will impact the current field of immuno-PET/SPECT.

#### 4.2.1. *^52^*Manganese

^52^Manganese (t_1/2_ = 5.6 days) is probably the most promising radionuclide entering the field of immuno-PET. ^52^Mn has suitable half-life for Ab imaging and a comparable positron branching ratio (29.7%) and energy (242 keV) as ^89^Zr (23%/ 396 keV). However, the radiometal is accompanied with rather high energy γ-rays (744 keV, 935 keV and 1434 keV) resulting in high radiation burden and a negative impact on the imaging quality [147].

Extensive comparison of ^52^Manganese complexation using different chelators is still absent, but DOTA could be easily radiolabeled at room temperature, neutral pH and <1 min. Follow-up in vitro stability tests showed intact tracer up to 2 days in bovine serum [148].

Up until now only one in vivo study using ^52^Mn-mAb has been performed. In this study an anti-CD105 Ab, [^52^Mn]Mn-DOTA-TRC105, was used to investigate angiogenesis in a 4T1 breast cancer xenograft model. The tumor could be visualized in this model with peak tumor uptake of 19% ID/g at 24 h pi (Figure 4).

Importantly, no in vivo instability of the [^52^Mn]Mn-DOTA-TRC105 complex could be observed. Remarkably, high bone uptake was present in [^52^Mn]Mn-DOTA-TRC105 but not in ^52^MnCl_2_ injected mice, which the authors explained as a direct interaction between bone and DOTA-bound manganese instead of bone seeking properties of ^52^Mn [149].

#### 4.2.2. Gallium

^68^Gallium (t_1/2_ = 67.71 min) has been extensively used in radio-pharmacy and made available worldwide due to the ^68^Ge/^68^Ga generator. Whilst successfully implemented in patients for immuno-PET imaging of nanobodies, its short half-life makes it unsuitable for full mAb imaging [16]. However, the isotopes ^66^Ga (PET) (t_1/2_ = 9.5 h) and ^67^Ga (SPECT) (t_1/2_ = 3.3 days) are more suited and have been investigated for mAb imaging, but their availability is not so straightforward. Gallium radiochemistry/labeling has been extensively studied due to the popularity of ^68^Ga providing a large advantage for ^66^Ga/^67^Ga immuno-PET/SPECT in animal models.

Numerous chelators can be complexed with gallium (see Tsionou et al. [150]). The different isotopes of gallium can be complexed and radiolabeled in a similar way. The ability to radiolabel gallium in mild conditions, low temperature and neutral pH, are of great importance in selecting the right chelator for immuno-PET/SPECT imaging, to avoid Ab degradation and denaturation. The chelator that has been specifically made for gallium radiolabeling using this purpose, with reduced cavity size compared to DOTA, is NOTA [150].

^66^Gallium has been successfully evaluated in vivo, with [^66^Ga]Ga-NOTA-TRC105 mAb able to image CD105 in 4T1 xenografts. Tumor uptake was 8.5% ID/g at 20 h pi [151]. Goethals et al. evaluated ^66^Ga-antimyosin Ab in two dogs and were able to image posterior wall necrosis in myocardial infarction [152]. However, clinical translation of this radiometal is hindered or even impossible because of high energy β^+^ emission and abundant high energy γ-rays (1039 (37%), 2752 (23%), and 4295 keV (4%)), which would result in poor imaging quality, reduced spatial resolution and high radiation burden for patients.

^67^Gallium proved to have potential for in vivo immuno-SPECT as shown by Milani et al. In their study [^67^Ga]Ga-DTPA-anti-Ror1 mAb was evaluated 4T1 xenografts and showed 6.5% ID/g tumor uptake [153]. Translation of [^67^Ga]Ga-DFO-anti-CEA-mAb to the clinic was even possible and showed detection of 86% of the primary tumor sites [154].

#### 4.2.3. *^90^*Niobium

^90^Niobium(t_1/2_ = 14.6 h) has been getting attention from the radio-pharmacy research community for Ab imaging, probably due to the suitable characteristics of the radiometal, especially the high positron branching ratio (51%) and acceptable positron energy (662 keV). The positron energy is more favorable compared to other less explored radionuclides (^86^Y, ^66^Ga, ^72^As) but higher than the conventional radioisotope ^89^Zr [155]. Some limitations are the high energy γ ray co-emission, which results in high radiation dose, decreased imaging quality and suboptimal half-life for Ab imaging, which is more suited for Ab fragments. However, the most important limitation is the ^90^Niobium production methods which are far from optimized and can contain long half-life co-produced Nb impurities due to the lack of enriched targets [156].

Since ^90^Nb-immuno-PET is still in an early stage, additional research is required to select more optimal chelators for mAb radiolabeling. An early complexation study between different chelators pointed at DFO as best suited chelator, within mild, Ab desired, reaction conditions [157]. This initial study prompted investigators to assess radiolabeled [^90^Nb]Nb-DFO-rituximab, which showed >90% yield after 1 h [158]. Radchenko et al. concluded these ^90^Nb-mAb radiolabeling experiments by assessing [^90^Nb]Nb-DFO-bevacizumab in a VEGF-transfected human breast cancer xenograft. Although tracer stability was good and tumor imaging feasible, only 3% ID/g uptake at 24 h pi was observed in the tumors, possibly due to the low specific activity of the [^90^Nb]Nb-DFO-rituximab [156].

#### 4.2.4. Arsenic

Arsenic is a biological reactive element with strong binding to proteins resulting in systemic toxicity with fatal systemic exposure estimated at 0.6 mg/kg daily. However, when investigated as radiometal isotopes subtoxic doses are sufficient for the use as imaging agent [159]. This high biological reactivity is the main benefit over other radiometals since Arsenic can bind directly to sulfhydryl groups of Abs, avoiding the use of chelators and therefore facilitating the radiolabeling procedure [160]. In addition, the intrinsic characteristics of the radioisotope are favorable with high positron branching ratio of ^72^As (t_1/2_ = 1.1 days, β^+^ 86%) and possible theragnostic ^74^As (t_1/2_ = 17.8 days, β^+^ 26%/ β^−^). A drawback of ^72^As is the high energy positrons resulting in decreased resolution and high radiation dose. In addition, production and supply of clinical grade ^72^As is questionable with only a few production sites available worldwide

In a first proof of concept study, Jennewein et al. modified Abs with N-succinimidyl S-acetylthioacetate (SATA), to introduce additional sulfhydryl groups onto the Ab. [^74^As]As-SATA-bavituximab was then evaluated in R3327-AT1 tumor rat xenografts showing promising tumor/muscle ratios, however at 72 h pi only 0.65% ID/g tumor radiotracer uptake could be observed ex vivo [161]. In more recent work Ellison et al. radiolabeled TRC105 mAb directly (by reducing Ab thiol groups using TCEP) and indirectly after introducing sulfhydryl groups via Traut’s reagent with ^72^As. In both approaches identical biodistribution with free ^72^As could be observed, showing rapid renal clearance and therefore confirming in vivo dearsenylation and concluding Traut’s reagent as suboptimal arsenic radiolabeling technique [162]. Currently, arsenic immuno-PET mainly suffers from in vivo dearsenylation, which demands the development of better suited chelators before additional immuno-PET research can be done. Alternative concepts have been introduced to solve these stability concerns using dithiol-containing dihydrolipoic acid or trithiol ligands, which resulted in the formation of stable complexes [162,163]. However, in current stage ^72^As/^74^As still has a long way to go before translation to clinic is even remotely possible.

## 5. Conclusions and Future Directions

Preclinical applications of immuno-PET/SPECT are being heavily investigated introducing new technologies and solutions to old problems. However, translation of different radiotracers to the clinic is somewhat hampered, mostly because of absent validation of immuno-PET/SPECT radiotracers in a specific disease setting. In our understanding, implementing immuno-PET/SPECT in clinical practice would result in personalizing medicine: evaluating target expression and therapeutic regimens, predicting response, improving benefit/cost ratio, and therefore optimizing patient care. This claim has been endorsed in numerous clinical studies using [^89^Zr]Zr-DFO-trastuzumab, [^89^Zr]Zr-DFO-bevacizumab, [^89^Zr]Zr-DFO-nivolumab and others. Another important application of immuno-PET/SPECT is the biopharmaceutical antibody development process. Implementation and evaluation of antibody radioimmunoconjugates in animal models and early phase clinical trials have the ability to reduce costs and boost effectiveness. For example, identification of unwanted biodistribution or enriching patient selection.

To conclude this review paper and to answer which strategies are the best to answer which strategies are the best when creating your antibody-PET/SPECT radiotracer is not that straightforward. In bioconjugation one you opt for randomly or site-specifically bioconjugates? Site-specific bioconjugation is undoubtably beneficial to produce homogenous conjugates and reduce batch to batch variability and therefore address the major concern of regulatory authorities. However, creating site-specifically immunoconjugates can be challenging in some strategies and require extensive know-how. In addition, when evaluating the benefit of site-specifically over randomly Ab bioconjugates regarding immunoreactivity, it seems from results discussed above that the impact is not that distinct in some approaches. Full mAbs contain large Fc regions and when your chelator is attached to this region impact on immunoreactivity is expected to be minimal. On the other hand, when using Ab fragments as your targeting vector immunoreactive impact will be more defined and these will benefit more from site-specific conjugation.

Is pretargeting then the way to go in selecting your immuno-PET/SPECT tracer? The pretargeting approach allows the introduction of short-lived radionuclides in immuno-PET/SPECT, which generally show better intrinsic properties (high positron branching ratio, low positron energies) than longer lived radioisotopes and additionally reduces the radiation dose exposure for subjects. However, internalization of the targeted vector reduces the amount of available moiety for radioligand binding and downstream radiotracer uptake in the tumor. In contrast, directly radiolabeled Ab internalization after binding generally results in tumor cell residualization of the radioactivity and therefore increased tumor imaging contrast.

What radioisotope is perfect for your application? This is by far the most difficult question to answer since all radioisotopes show benefits and limitations as discussed above. Generally, the half-life of the radionuclide and biological vector (Ab) should match in decay and clearance respectively (^89^Zr, ^124^I, ^111^In), but perhaps shorter-lived radionuclides show better intrinsic characteristics for your application (^64^Cu, ^74^As theragnostic). The initial proof of concept of some of the emerging radionuclides (^90^Nb, ^52^Mn) show great results and can perhaps have a bright future in immuno-PET. Unfortunately, the current major concern is their availability and purity, which explains their under investigated application potential. Several other, conventional radionuclides (^124^I,^89^Zr,^64^Cu,^99m^Tc,^111^In) have been made available worldwide with clinical grade application potential. The intrinsic characteristics are also important in selecting your radionuclide. In PET, high positron branching ratio (^72^As, ^76^Br, ^90^Nb), low positron energy (^89^Zr, ^74^As, ^64^Cu, ^52^Mn, ^90^Nb) and low energy γ emission (^64^Cu) are required for optimal image quality. In SPECT imaging single photon emission radionuclides (^99m^Tc, ^111^In) are desired over multiple high energy emission radionuclides (^123^I, ^67^Ga). A final aspect that has to be considered is the radiation exposure, which is highest in long lived radio-isotopes with high positron energy and γ emission. This may represent an important aspect that could result in failure to acquire approval of Ab-PET/SPECT tracer by the regulatory authorities. However, in radiolabeled Ab fragments this is less of a problem since these are radiolabeled with short half-live radioisotopes and undergo faster clearance, but accumulation in the kidneys can result in renal toxicity. In addition, introducing new generation PET/SPECT-scanners with improved sensitivity can overcome the issue of radiation exposure for regulatory authorities and facilitate the use of immuno-PET/SPECT in the upcoming years.

This review paper showed the remarkable possibilities and successes that immuno-PET/SPECT has achieved over the past decades. While breakthrough applications in routine clinical care are currently still pending, we are convinced that this will change in the upcoming years.

## Figures and Tables

**Table 1:** Overview of radionuclides’ decay properties, emission and chelators for monoclonal antibody immuno-PET/SPECT imaging [81].

Radionuclides	Decay-Life	Production Method [82] ***	Emission	Labeling Strategies
**PET**			**β^+^ (%; E_Mean_)**	
^124^Iodine	4.2 days	Cyclotron	β^+^ (12%; 687 keV) β^+^ (11%; 975 keV)	Direct radiolabeling
^89^Zirconium	3.3 days	Cyclotron	β^+^ (23%; 396 keV)	DFO, DFO*, DFOSq, HOPO
^72^Arsenic	1.1 days	Generator	β^+^ (6%; 824 keV) β^+^ (64%; 1117 keV) β^+^ (16%; 1528 keV)	Direct labelling, SATA, Traut’s reagent
^74^Arsenic	17.8 days	High energy Cyclotron	β^+^ (26%; 408 keV) β^−^	Direct labelling, SATA, Traut’s reagent
^64^Copper	12.7 h	Cyclotron	β^+^ (18%; 278 keV) β^−^	NOTA
^86^Yttrium	14.7 h	Cyclotron	β^+^ (32%; 394–1437 keV) *	DOTA, CHX-A″-DTPA
^76^Bromine	16.2 h	Cyclotron	β^+^ (55%; 336–1800 keV) *	Direct radiolabeling
^52^Manganese	5.6 days	High energy cyclotron	β^+^ (29%; 242 keV)	DOTA
^90^Niobium	14.6 h		β^+^ (51%; 662 keV)	DFO
^66^Gallium	9.5 h	Cyclotron	β^+^ (4%; 397 keV) β^+^ (51%; 1904 keV)	NOTA
**SPECT**			γ (%; Energy) **	
^123^Iodine	13.2 h	High energy Cyclotron	γ_1_ (83%; 159 keV) γ_2_ (1%; 529 keV)	Direct radiolabeling
^131^Iodine	8.0 days	Reactor	γ_1_ (82%; 364 keV) γ_2_ (7%; 637 keV)	Direct radiolabeling
^67^Gallium	3.3 days	High energy Cyclotron	γ^1^ (3%; 91 keV) γ^2^ (39%; 93 keV) γ^3^ (21%; 184 keV) γ^4^ (2%; 209 keV) γ^5^ (17%; 300 keV) γ^6^ (5%; 394 keV)	NOTA
^99m^Technetium	6 h	Generator	γ (89%; 141 keV)	HYNIC, N_2_S_2_ ligand
^111^Indium	2.8 days	High energy Cyclotron	γ^1^ (91%; 171 keV) γ^2^ (94%; 245 keV)	DOTA, CHX-A″-DTPA

* Range different Eβ+Average /** Gamma rays used solely for SPECT imaging/ *** High energy cyclotron >20 MeV proton energies.
